# Antibiofilm Activity of Epinecidin-1 and Its Variants Against Drug-Resistant *Candida krusei* and *Candida tropicalis* Isolates from Vaginal Candidiasis Patients

**DOI:** 10.3390/idr16060096

**Published:** 2024-12-12

**Authors:** Sivakumar Jeyarajan, Sukumar Ranjith, Raja Veerapandian, Kalimuthusamy Natarajaseenivasan, Prahalathan Chidambaram, Anbarasu Kumarasamy

**Affiliations:** 1 Microbial Biotechnology Laboratory, Department of Marine Biotechnology, Bharathidasan University, Tiruchirappalli 620024, India; jeyaraja@umich.edu (S.J.); starsukumar1996@gmail.com (S.R.); 2 Transgenic Animal Model Core, Biomedical Research Core Facilities, University of Michigan, Ann Arbor, MI 48109, USA; 3 Center of Emphasis in Infectious Diseases, Department of Molecular and Translational Medicine, Paul L. Foster School of Medicine, Texas Tech University Health Sciences Center El Paso, El Paso, TX 79905, USA; r.veerapandian@ttuhsc.edu; 4 Department of Microbiology, Bharathidasan University, Tiruchirappalli 620024, India; director-rmrcne@icmr.gov.in; 5 ICMR-Regional Medical Research Centre, Lahowal, Dibrugarh 786010, India; 6 Department of Biochemistry, Bharathidasan University, Tiruchirappalli 620024, India; prahalath@bdu.ac.in

**Keywords:** antimicrobial peptides, epinecidin-1, multi drug resistance, *Candida krusei*, *Candida tropicalis*, antifungal peptides, biofilm inhibition, scanning electron microscopy

## Abstract

**Background/Objective:** Indwelling intrauterine contraceptive devices (IUDs) have surfaces that facilitate the attachment of *Candida* spp., creating a suitable environment for biofilm formation. Due to this, vulvovaginal candidiasis (VVC) is frequently linked to IUD usage, necessitating the prompt removal of these devices for effective treatment. In this study, we evaluated the susceptibility of antimicrobial peptides in vitro against biofilm forming, Amphotericin B (MIC50 > 2 mg L^−1^) resistant *Candida krusei* and *Candida tropicalis* isolated from IUD users who had signs of vaginal candidiasis (hemorrhage, pelvic pain, inflammation, itching, and vaginal discharge). Three antimicrobial peptides, namely, epinecidin-1 (epi-1) and its two variants, namely, variant-1 (Var-1) and variant-2 (Var-2), which were reported to have enhanced antibacterial activity were tested against IUD isolates (*C. krusei* and *C. tropicalis*) with pathogenic form of *Candida albicans* as control. Variants of epi-1, namely, Var-1 and Var-2 were created by substituting lysine in the place of histidine and alanine. **Methods:** The antimicrobial activity was measured using the microbroth dilution method to determine the minimum inhibitory concentration (MIC) of peptides against *C. albicans*, *C. krusei* and *C. tropicalis*. The MIC of each peptide was used for biofilm assay by Crystal violet staining, Scanning Electron Microscopy, and Reactive Oxygen Species (ROS) assay. To find the possible mechanism of anti-biofilm activity by the peptides, their ability to interact with *Candida* spp. cell membrane proteins such as Exo-β-(1,3)-Glucanase, Secreted Aspartic Proteinase (Sap) 1, and N-terminal Domain Adhesin: Als 9-2 were determined through PatchDock. **Results:** The MIC values of peptides: epi-1, var-1 and var-2 against *C. albicans* are 128 μg mL^−1^, 64 μg mL^−1^ and 32 μg mL^−1^, *C. tropicalis* are 256 μg mL^−1^, 64 μg mL^−1,^ and 32 μg mL^−1^ and *C. krusei* are 128 µg mL^−1^, 128 µg mL^−1^ and 64 µg mL^−1^, respectively. Both the variants outperformed epi-1. Specifically for tested *Candida* spp., var-1 showed two- to four-fold enhancements and var-2 showed two- to eight-fold enhancements compared to epi-1. Electron microscopy confirmed that the mechanism of action involves pore formation thus inducing reactive oxygen species in *Candida* spp. cell membrane. Computational analysis showed that the peptides have a high tendency to interact with *Candida* spp. cell membrane proteins such as Exo-β-(1,3)-Glucanase, Secreted Aspartic Proteinase (Sap) 1, and N-terminal Domain Adhesin: Als 9-2, thereby preventing biofilm formation. **Conclusions:** The in vitro evidence supports the potential use of epi-1 and its variants to be used as an anti-biofilm agent to coat IUDs in the future for therapeutic purposes.

## 1. Introduction

Candidiasis, or *Candida* spp. infections, has emerged as a critical global health concern in recent decades due to their increased resistance to currently available antifungal drugs such as fluconazole, echinocandins, and polyenes [1,2]. According to the CDC, Candidemia accounts for 25% of mortality among the patients hospitalized due to implanted device-related bloodstream infections [1].

Intrauterine devices are cost-effective and one of the most popular methods of contraception worldwide. However, the presence of these devices provides a solid surface for the attachment of commensal microorganisms such as bacteria and *Candida* spp., serving as an ideal niche for biofilm formation. This biofilm can lead to an exponential increase in their numbers and transform the commensals into pathogens. In this instance, IUDs serve as a reservoir of candida infection, accumulating larger biofilm mass and contributing to recurrent infection [2]. Due to this pathogenicity, inflammation arises in the tissues around the device, leading to disease and a weakened immune system. Based on the symptoms, if drugs are prescribed and not taken in the prescribed manner, it causes drug resistance. The growth of drug-resistant *Candida* spp. on these devices progresses vaginal candidiasis and alarms users, leading to a loss of confidence. Vulvovaginal candidiasis can enter the bloodstream and become invasive, causing candidemia, which is difficult to treat and may result in multi-organ failure. Thus, the progression from using a contraceptive device to a life-threatening condition is alarming and often obscured. While amphotericin B is the last resort for antifungal treatment, it is generally not prescribed for vaginal candidiasis to avoid resistance development. Unfortunately, amphotericin B resistance has also been observed. In cases of resistance, physicians often increase the dose of amphotericin B, which is toxic to humans and causes further complications for already ill patients. The World Health Organization (WHO) further reports that immunocompromised intrauterine device users are at an elevated risk [3]. The high mortality rate associated with these infections underscores the urgent need for effective infection control measures and highlights the urgent need for effective anti-*Candida* treatments.

The multidrug resistance mechanisms in *Candida* spp. pose a significant challenge, particularly because of their unique outer membrane chemical composition. *Candida* spp. possess a defence-oriented outer membrane that consists of specialized lipids and carbohydrates. They can alter this structure to evade the impact of drugs, especially the echinocandins class of drugs that have a binding target on cell wall carbohydrates. For example, *Candida* spp. can mutate the enzyme (1,3)-β-D-glucan synthase, which modifies the carbohydrate moiety in their cell wall preventing echinocandins from disrupting the cell wall. Another class of drugs, azoles, acts on the fungal cell membrane by inhibiting lanosterol 14-α-demethylase (Erg11) [4]. *Candida* has an in-built mechanism to upregulate the efflux transporter pumps like ABC (ATP-binding cassette) superfamily transporters to efflux the azole drugs. Furthermore, the polyene category drug: Amphotericin B, forms aggregates outside cell membranes that extract ergosterol from fungal cell membranes, functioning like a sterol “sponge.” Although resistance is very rare, it can develop through mutations in the genes responsible for ergosterol production, leading to a reduction in ergosterol and accumulation of other sterols to maintain the integrity of the membrane. Additionally, *Candida* spp. undergoes a transformation process, often referred to as hyphal transformation [5], which assists them in evading the host’s immune system. These traits pose additional difficulties in orchestrating a successful treatment strategy for *Candida* infections, necessitating new therapeutics that can overcome the above problems.

Recent research has concentrated on the study of cationic antimicrobial peptides (AMPs), which exhibit toxicity to *Candida* spp. while sparing normal mammalian cells [6,7] because the membrane properties and composition of *Candida* spp. differ from those of mammalian cells. AMPs consist of repeating sequences of positively charged and hydrophobic amino acids, endowing them with an amphiphilic nature. This amphiphilic property enables AMPs to bind to the negatively charged *Candida* spp. membrane (contributed by phosphatidyl inositol and phosphatidic acid) and penetrate through the hydrophobic membrane, forming channels that cause cytosolic content leakage. Because AMPs target negative charges rather than specific carbohydrate or lipid moieties, the likelihood of resistance is low. Hence, AMPs are considered potential alternatives to conventional *Candida*-cidals. Currently, there are more than 1500 AMPs with antifungal action in the APD3 database https://aps.unmc.edu/ (accessed on 8 December 2024) [8]. A few examples of AMPs having significant anti-*Candida* properties are Histatins [9], protonectin [10], cathelicidins from chicken namely CATH-2 and LL-37 in humans [11], N-terminal domain of bovine lactoferrin [12], protegrin, etc.

Epinecidin-1, a cationic antimicrobial peptide (AMP) initially identified in grouper fish (*Epinephelus coioides*), displays a wide range of activity against bacteria, fungi, viruses, and protozoa [8,9,10,11,12]. As this peptide manifests such a broad spectrum of activity, we have further augmented its structural and functional stability through the substitution of lysine at the histidine and alanine residues, as reported in our previous study [10]. Incorporating lysine into peptides increases their cationicity and tendency to form alpha-helices. The periodic presence of lysine at certain intervals imparts amphiphilicity, which enhances antimicrobial activity. Since *Candida* spp. cell wall properties and characteristics are analogous to the peptidoglycan of bacterial cell walls, it prompted us to explore the anti-Candida potential of epinecidin-1 and its variants. In doing so, these variant peptides could possibly perform dual functions, serving as both antibacterial and anti-Candida agents. Our research findings reveal that these customized variants exhibit an increased Candida-cidal impact, like their bactericidal effects. The experimentally determined in vitro levels of susceptibility (MIC), crystal violet staining, and SEM confirm that all three peptides have antifungal potency with the variants exhibiting stronger activity.

## 2. Materials and Methods

### 2.1. Candida Strains, Peptides and Chemicals

Vulvovaginal candidiasis (VVC) isolates: Biofilm-forming *C. tropicalis* (strain CA4) and *C. krusei* (strain CA54) which were reported by Shanmugapriya et al. [13] were used for this study. Shanmugapriya et al. [13] studied the prevalence of non-albicans *Candida* (NAC) infections in IUD users and collected their endocervical swabs; women aged 20–35 years who had signs of pelvic inflammation, pruritus, hemorrhage, and vaginal discharge, etc. They characterized the prevalence of non-albicans *Candida* spp. (NAC) grown from endocervical swabs by CHROM agar and identified that twenty-three clinical *NAC* isolates (10 *C. krusei* and 13 *C. tropicalis*) were the most pervasive non-albicans *Candida* (NAC). Among them, *C. tropicalis* (strain CA4) and *C. krusei* (strain CA54) were found to be resistant to Amphotericin B with high biofilm-forming potential containing several microcolonies. The pathogenic strain of *C. albicans* obtained from the Microbial Type Culture Collection (MTCC 227) was used as a control test organism. Antimicrobial peptide epinecidin-1 and its lysine substituted variants namely *variant-1, and variant-2 were purchased from GenicBio Limited, Shanghai, China, which were synthesized using 9-fluorenylmethoxycarbonyl (Fmoc) solid-phase peptide synthesizer. The above-mentioned *Candida* spp. were grown in Sabouraud Dextrose broth (Millipore Sigma Burlington, Burlington, MA, USA). All additional chemical reagents employed in the testing procedures were of analytical grade.

### 2.2. Variants of Epinecidin-1

Epinecidin-1 and its lysine-replaced variants were tested for anti-*Candida* properties. Their sequences as reported in [14] with their corresponding site-specific replaced amino acids are shown in Table 1.

### 2.3. Antifungal Assay

Antimicrobial activity of the peptides (epi-1, var-1, and var-2) against *C. tropicalis* (strain CA4), *C. krusei* (strain CA54), and *C. albicans* (MTCC 227) was tested with the growth media: Sabouraud dextrose broth (SDB). The microbroth dilution method was used in accordance with the Clinical and Laboratory Standards Institute (CLSI) M27-Ed4 guidelines for antifungal susceptibility testing [15]. The stock solution of the peptide was diluted with phosphate-buffered saline (PBS) to reach concentrations of 1, 2, 4, 8, 16, 32, 64, 128, 256 and 512 μg mL^−1^ [5]. Aliquots (20 μL) from each dilution were distributed to a 96-well polystyrene microtiter plate, and each well was inoculated with 180 μL suspension of *Candida* spp. in SDB containing 1 × 10^6^ cells. Cultures were grown with gentle shaking for 24 h at 37 °C. A well containing only cells without peptides was used as growth or positive control and the plain broth was used as sterility or negative control. The absorbance was evaluated at 595 nm using a microplate reader (Bio-Rad, Hercules, CA, USA) and represented in terms of percentage of growth using growth control value as 100% growth. The minimal inhibitory concentration (MIC) of the peptides was defined as the lowest concentration at which the percentage of growth less than 15% was observed. The antimicrobial assay was carried out in triplicates and the growth percentage was plotted as mean ± S.D. Tukey’s multiple comparisons test (two-way ANOVA) was performed for statistical analysis and significance representations are ns, non-significant; * *p*  <  0.5, ** *p*  <  0.1, *** *p*  <  0.01, and **** *p*  <  0.001.

### 2.4. Biofilm Assay

To measure the ability of the peptides to inhibit the formation of biofilm, 1 mL suspension of *Candida* spp. (1 × 10^6^ cells) in SDB media were seeded into each well of a 24-well plate containing microscope coverslip ((circular borosilicate cover glasses (15 mm), Fisher Scientific, Hampton, NH, USA)) inserts [5]. The cover glass served as a substratum for microbial attachment. Peptides at their MICs were treated for 24 h. For *C. albicans*, 128 μg mL^−1^ of epi-1, 64 μg mL^−1^ of var-1, and 32 μg mL^−1^ of var-2 were used. For *C. tropicalis*, 256 μg mL^−1^ of epi-1, 64 μg mL^−1^ of var-1, and 32 μg mL^−1^ of var-2 were used. For *C. krusei*, 128 µg mL^−1^ of epi-1 and var-1, and 64 µg mL^−1^ of var-2, respectively, were used. After incubation, the spent media was aspirated and 1 mL of 0.1% (*w*/*v*) crystal violet dissolved in PBS was added to each well and incubated for 30 min to stain the cells. Post-staining, excess crystal violet was removed by washing twice with PBS. The cells were fixed with 4% formaldehyde, washed with PBS, and dehydrated with 80% (*v*/*v*) ethanol. The stained cover slips were examined under a light microscope with 40 × magnification. The cell numbers in each field were calculated using imageJ: region of interest (ROI) tool [16,17]. Since the biofilm layer contains clusters, manual discretion was applied to select individual cells, and the clusters were marked as a group of cells. The quantitated cells from peptide-treated fields (three different fields) were plotted as a graph. The ROI output from imageJ is shown in Supplementary Figure S1.

### 2.5. Scanning Electron Microscopy (SEM)

To view the morphological changes in *C. albicans* cells (MTCC 227) after treatment with epinecidin-1 and its variants, SEM was employed [18]. In brief, *C. albicans* cells were grown to a logarithmic phase with an inoculum size of (1 × 10^6^ cells) on a microscope cover slip which was inserted into each well of a 24-well dish. *C. albicans* were treated with epinecidin-1, var-1, and var-2 at their MIC of 128 μg mL^−1^, 64 μg mL^−1^ and 32 μg mL^−1^. Cells grown without any peptide were as negative control. After incubation for 6 h at 37 °C, the cells were washed twice with phosphate-buffered saline (PBS) pH 7.4, and metabolically fixed with an equal volume of 5% (*v*/*v*) glutaraldehyde at 4 °C overnight. The metabolically fixed cells were dehydrated with a serial gradient of ethanol wash from 70% to 100%. The coverslips were then sputter coated and examined under a scanning electron microscope (VEGA3 TESCAN, Kohoutovice, Czech Republic).

### 2.6. Molecular Docking Study

The Protein Data Bank (PDB) files of *Candida* exo-beta-(1,3)-glucanase (1CZ1), Sap1 (2QZW), and N-terminal domain of Als 9-2 (2Y7L) were obtained from the Research Collaboratory for Structural Bioinformatics (RSCB) protein data bank. The tertiary structure of the epinecidin-1 and designed variant peptides were obtained using i-TASSER [19]. The structures with minimum energy were selected for docking interaction using PatchDock [20]. The PatchDock output files were loaded into BIOVIA Discovery Studio (Dassault Systèmes, San Diego, USA) for representation of the amino acids interacting with the *Candida* cell wall proteins and antimicrobial peptides. The amino acids interacting within a 3Å distance are shown.

### 2.7. Measurement of Cellular ROS Production

Endogenous amounts of ROS were measured by fluorometric assay with 2′,7′-dichlorofluorescin diacetate (DCFHDA) as described in [10]. Briefly, *Candida* spp. (1 × 10^6^ cells) were seeded into 24 well polystyrene plates and treated with or without peptides at their respective MIC for 24 h. After 24 h, the cells were incubated with 10 μM of DCFHDA for 1 h and washed with PBS pH 7.4. They were then visualized in a fluorescent microscope (Accu-Scope, EXI-310, Commack, NY, USA) at 10× magnification and documented with green channel fluorescence intensities (excitation 488 nm and emission 525 nm, respectively). Hoechst dye was used to stain the nucleus. The number of cells in the field was quantitated using imageJ: as described in the manual (https://imagej.net/ij/docs/pdfs/ImageJ.pdf (accessed on 8 December 2024). Briefly, the background was subtracted, and the fields were made binary. Using the threshold, to differentiate the background versus cell spots, the number of cells in the fields was calculated. The number of cells in the blue and green channels was quantitated and the percentage of ROS-positive cells was plotted. The binary image output of blue and green channels from imageJ are shown in Supplementary Figure S2.

## 3. Results

### 3.1. Candidacidal Activity of Epinecidin-1 and Its Variants

Numerous studies, following thorough examination and biological characterization, have shown that increasing the cationicity and α-helicity of antimicrobial peptides (AMPs) significantly boosts their antimicrobial activity [5,14,21,22,23,24,25]. In our previous reports, we have shown that substituting lysine in epinecidin-1 has resulted in improved structural stability and enhanced antibacterial potency of variant-1 and variant-2.

**Figure 1:**
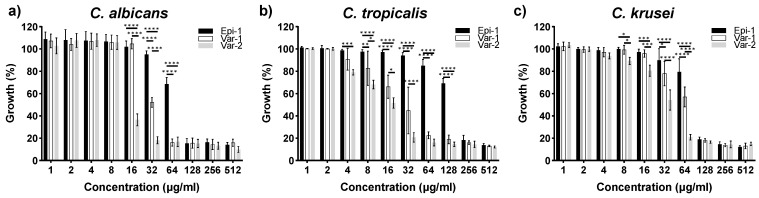
Susceptibility of the peptides against (**a**) *C. albicans* (MTCC 227), (**b**) *C. tropicalis* (CA4) and (**c**) *C. krusei* (CA54). Epi-1, var-1, and var-2 inhibited growth of *C. albicans* (MTCC 227) at 128 μg mL^−1^, 64 μg mL^−1^ and 32 μg mL^−1^. For *C. tropicalis;* epi-1, Var-1 and Var-2 showed MIC of 256 μg mL^−1^, 64 μg mL^−1^, and 32 μg mL^−1^, respectively. For *C. krusei*; epi-1 and Var-1 showed MIC of 128 μg mL^−1^ and variant-2 showed MIC of 64 μg mL^−1^. Cell density was measured at 595 nm after 24 h. Growth of the cells measured without peptides is used as a control with 100% growth. The level of significance was measured for variants compared to epi-1 at their respective concentrations tested. Statistical value representations are * *p*  <  0.5, ** *p*  <  0.1, *** *p*  <  0.01, and **** *p*  <  0.001.

*Candida*-cidal activity of epinecidin-1 and its variants against the VVC isolates were studied using the broth dilution method to quantitatively determine their growth, as measured by optical density at 595 nm. Figure 1 shows the plot of growth of the isolates against the range of concentration (1 to 512 µg mL^−1^) tested with the peptides. The percentage of growth was measured by normalizing with the cells grown without peptides, which were considered 100%. The minimum inhibitory concentration (MIC) for the peptides was determined from the lowest concentration at which the peptides were able to inhibit the growth of the VVC isolates. 

For *C. albicans*, the MIC values for epi-1, var-1, and var-2 are 128 μg mL^−1^, 64 μg mL^−1^ and 32 μg mL^−1^, respectively. The variants exhibited significant growth inhibition in the concentration range of 16 to 32 μg mL^−1^. *C. tropicalis* showed similar patterns of sensitivity, with MIC values of 256 μg mL^−1^, 64 μg mL^−1,^ and 32 μg mL^−1^ for epi-1, var-1, and var-2, respectively. For *C. krusei*, the MIC values of epinecidin-1 and variant-1 are 128 µg ml^−1^ and for variant-2 it is 64 µg ml^−1^ respectively. For the variants against *C. tropicalis* and *C. krusei*, the variants show growth inhibition in the range of 4 to 128 μg mL^−1^. At higher concentrations, all three peptides inhibited the growth of *Candida* spp. to the maximum extent. Among the *Candida* spp. studied, the variants performed better than epi-1 having the lowest MIC values.

### 3.2. Epinecidin-1 and Its Variants Inhibit Biofilm Formation of VVC Isolates

After determining the MIC, the ability of the peptides to prevent the biofilm formation of VVC isolates formed on glass coverslips was determined by crystal violet staining. As these isolates were reported to form biofilm, they were cultured with and without the peptides (at their respective MIC values) for a period of 24 h. After the treatment period, the coverslips containing *Candida* spp. cells were stained with crystal violet and then examined under an inverted 40 × light microscope.

**Figure 2:**
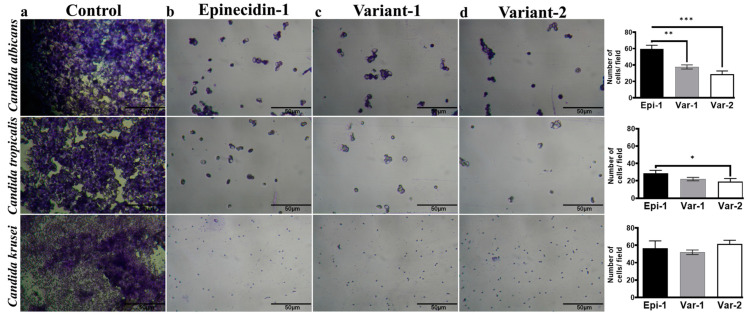
Light microscopic image of crystal violet stained *C. albicans* (MTCC 227), *C. tropicalis* (CA4), and *C. Krusei* (CA54) are shown in individual rows at 40× magnification. The columns represent (**a**) control, (**b**) epinecidin-1, (**c**) variant-1, (**d**) variant-2. The quantitative plots for each row is provided in the right column, The cells were treated with peptides at their respective MIC and incubated for 24 h. The image panel shows the effects of peptides in reducing biofilm formation. On the right, there are bar graphs representing the quantitative analysis of the number of cells under each treatment condition for each species. The quantitative cell number of the control is provided in the text but is not displayed in the plot because of variation in the Y-axis scale. Peptide treatment has invariably decreased the *Candida* spp. Cell numbers compared to the control. Statistical significance representations are * *p*  <  0.5, ** *p*  <  0.1, and *** *p*  <  0.01.

The images shown in Figure 2, provide visible evidence of the peptide’s impact on biofilm formation. Control samples (*Candida* spp. cells without peptides) showed dense cell clusters indicative of substantial biofilm. Conversely, the peptide-treated cover glass did not have a dense biofilm matrix and showed a significant reduction in cell numbers. The number of cells present in each field was calculated using imageJ. For the controls, the average number of countable cells in the field of *C. albicans* is 726 ± 45, *C. tropicalis* are 716 ± 174 and *C. krusei* are 1433 ± 108. Some patches in the fields show dense clusters of cells, so they could not be counted. Hence, they are more in numbers than the scores mentioned. For the peptides, each of them has reduced the cell numbers by a factor of 10 compared to the control. For *C. albicans*, the variants have outperformed epi-1 significantly. Regarding *C. tropicalis*, var-2 showed a significant reduction in cell numbers compared to epi-1 and var-1. The cell numbers pertaining to the peptide-treated fields of *C. krusei* were similar. This prevents the seeding potential of *Candida* spp. from forming a biofilm on the surface.

### 3.3. Epi-1 and Its Variants Disrupt Candida albicans Membrane Integrity

**Figure 3:**
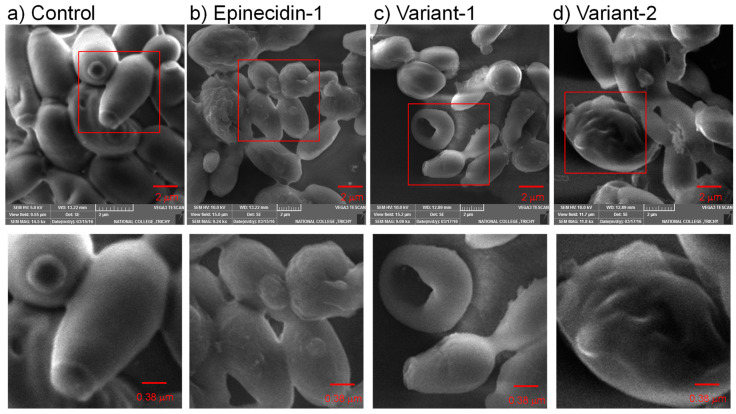
Scanning electron microscopic (SEM) image of *C. albicans* (MTCC 227) (**a**) Control, and (**b**–**d**) peptide-treated cells. The cells were treated with the peptides at their respective MIC and incubated for 6 h. The reduction in the number of cells noted as empty spaces between the cells and the disruption of the cell membrane demonstrates the membrane disruption activity of the peptides. The peptide-treated cells show an aberrant surface with internally collapsed morphology due to cytoplasm leakage and appear rugged. Bottom panel shows an enlarged view of the red inset box marked in the upper panel.

Scanning Electron Microscopy (SEM) was utilized to investigate the impacts of epi-1 and its variants on membrane penetration efficiency in the *C. albicans* cells as illustrated in Figure 3. Under SEM, the untreated control *C. albicans* cells exhibited typical features: oval shapes, smooth surfaces, polar buds, and bud scars. For examining the effects of the peptides on the *C. albicans* membrane, cells were incubated with 1 × MIC of the peptides for 6 h. The 6 h time point was chosen because a 24 h treatment typically detached all the cells from the cover slips. Since the peptide made pores on the membrane prolonged treatment with the peptides did not permit good sputtering of the heavy metal on the perforated cell surface.

The SEM images indicate that the peptide treatment altered the external cell morphology of *C. albicans*. When treated with epinecidin-1, the cells no longer appeared as smooth as the untreated cells; the membrane seemed to inflate with a rough, uneven surface. The size of the cells is also small possibly due to cytoplasmic leakage (Figure 3b inset). Additionally, numerous vesicular structures were observed surrounding the treated cells. Regarding the var-1 peptide, the cells displayed signs of shrinkage, potentially due to a loss of cytosolic content. Substantial perforations (Figure 3c inset) were apparent, suggesting the peptide-induced damage permeates the membrane, and more rounded cells were observed compared to the elongated cell morphology in the control. Pertaining to var-2, the cells endured severe damage, leading to the collapse of the cell wall and a noticeably bloated appearance, suggesting the possibility of a large pit on the opposite side. These findings show strong evidence of the antimicrobial activity of epinecidin-1 and its variants against *C. albicans*, resulting in significant membrane penetration potential and destabilization of the cell membrane.

### 3.4. Molecular Interaction of Peptides with Candida spp. Membrane Proteins

**Figure 4:**
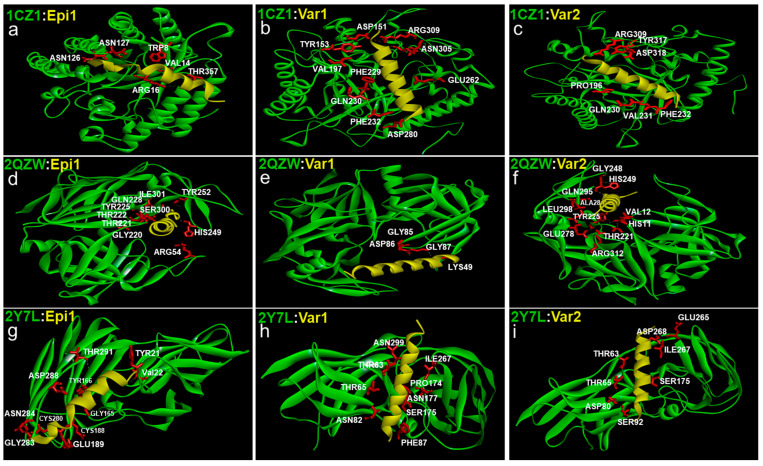
Docking interactions of epinecidin-1 and its variants with *Candida* Exo-B-(1,3)-Glucanase: PDB ID 1CZ1 (**a**–**c**), Secreted aspartic proteinase (Sap) 1: PDB ID 2QZW (**d**–**f**) and N-terminal domain of Als 9-2: PDB ID 2Y7L (**g**–**i**) biofilm forming membrane receptors. The *Candida* cell wall proteins are represented green in colour and the peptides are yellow in colour. The amino acids originating from the *Candida* cell wall proteins interacting with the peptides are represented in white with their three-letter code and their corresponding position. The amino acid side chain of the *Candida* protein (TYR, VAL, GLY, TYR, CYS, GLU, CYS, GLY, ASN, ASP, THR) acting as a binding pocket for the peptides is shown red in colour.

To determine the capability of antimicrobial peptides to interact with the biofilm-forming proteins in the cell walls of *Candida* spp., computational methods such as PatchDock and BIOVIA were used. The interface area of the peptide binding to three specific *Candida* spp. cell wall proteins: (a) Exo-B-(1,3)-Glucanase, (b) Secreted Aspartic Proteinase (Sap) 1, and (c) N-terminal domain of *C. albicans* adhesin: Als 9-2 (Agglutinin Like Sequence Super Family 9; allelic isoform 2) were calculated and portrayed in Table 2. Data from PatchDock suggested that all the peptides bound to all the three selected target *Candida* cell wall proteins within a 3Å space and are represented in Figure 4. The amino acids of the *Candida* cell wall proteins interacting with the peptides are shown in white in Figure 4 and enumerated in Supplementary Table S1.

A larger interface area signifies more interaction of the *Candida* spp. cell wall proteins with the peptides. For Exo-B-(1,3)-Glucanase, var-1 covers a larger interface area followed by var-2 and epinecidin-1. The high positive charge of var-1 and var-2 leads to them having more interaction with the negatively charged amino acids of Exo-B-(1,3)-Glucanase. Var-1 also interacts with hydrophobic and aromatic amino acids. Through Van Der Waals forces, Epinecidin-1 can interact with positively charged amino acids, while also binding with aromatic and hydrophobic amino acids like valine, proline, phenylalanine, and glutamine.

In terms of interaction with secreted aspartic proteinase, var-2 exhibits a greater interface area, followed by epinecidin-1 and var-1. Since var-2 has a more positive charge, its interaction with SAP is more profound. All three peptides interact with unique negatively charged amino acids and demonstrate interactions with hydrophobic and aromatic amino acids.

Examining the *Candida* spp. adhesin protein, namely (N-terminal domain of Als 9-2), var-1 presents a higher interface surface area, while epinecidin-1 and var-2 show similar values of interface area. Both var-1 and var-2 share mutual interactions with threonine 63, threonine 65, serine 175, and isoleucine 267 via hydrophobic and charge-based interactions. Most of the interactions for var-2 occur through charge-based interactions. Meanwhile, in addition to these common interactions, variant-1 uniquely engages with asparagine using Van Der Waals forces. Epinecidin-1 exhibits a higher number of binding contacts with hydrophobic amino acids and fewer with both negatively and positively charged amino acids. These interactions primarily involve oppositely charged electrostatic interactions and likely charged Van Der Waals interactions. The binding site is also different from variants 1 and 2.

Overall the positive charge amino acids in the variants have the strongest interactions with the negative and hydrophobic patches of the membrane proteins.

### 3.5. Epinecidin-1 and Its Variants Induced ROS Production

**Figure 5:**
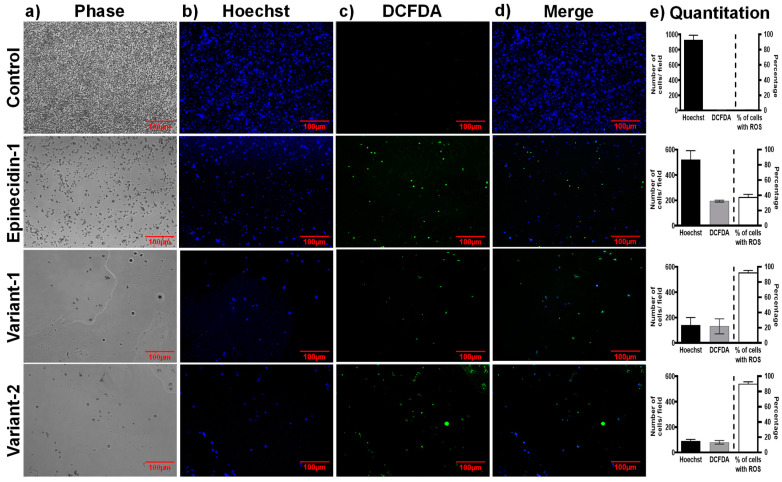
Effect of epinecidin-1 and its variants on the production of ROS by in *C. albicans*. Column (**a**) control cells, (**b**) epi-1, (**c**) var-1, and (**d**) var-2 peptide-treated cells and (**e**) Quantitation of number of cells in the field. Logarithmically growing *C. albicans* cells (1 × 10^5^) were pre-incubated with 20 μM DCFHDA and grown for 24 h at 37 °C. Cells were treated with and without peptides. After 24 h, the culture dish was washed with phosphate-buffered saline pH 7.4 and observed under a fluorescent microscope. Hoechst staining was performed alongside to determine the number of live cells. The images were captured at an excitation wavelength of 488 nm and an emission wavelength of 525 nm. The bar graphs on the right show the quantitation of cells in blue and green channels. The bar on the extreme right shows the percentage of cells with ROS calculated from the number of cells in the blue and green channels. The control does not have ROS-positive cells. The variants have more than 90% ROS production.

To determine whether the peptides have the ability to induce production of reactive oxygen species (ROS) production in *Candida* spp. we used dichlorofluorescein diacetate (DCFDA) after treatment with epinecidin-1 and its variants. DCFDA is a cell-permeant dye that is oxidized to yield fluorescence when exposed to ROS. The fluorescence can be monitored with the excitation wavelength of 488 nm and the emission wavelength of 525 nm. In Figure 5, the control cells (no treatment) showed no DCFDA fluorescence. The cells treated with the peptides for 24 h showed strong DCFDA fluorescence intensity. Epi-1 treated cells show 40% of cells with ROS. Var-1 and var-2 show more than 90% of ROS-positive cells indicating that the variants are effective in generating ROS compared to epi-1.

## 4. Discussion

Vulvovaginal candidiasis (VVC) is a common condition in women, causing vaginal discharge and pruritus, with 70–75% of women [26,27] experiencing VVC at least once during their reproductive years. The incidence of vaginitis caused by non-albicans Candida (NAC) species is increasing, posing significant challenges due to their reduced susceptibility to fluconazole, a commonly used treatment. NAC infections are often associated with recurrent disease, and the diagnosis of VVC is frequently made based on non-specific symptoms and treated without culture confirmation. This can lead to the development of antifungal resistance. The widespread use of oral fluconazole for prophylaxis, especially in immunocompromised patients such as those with HIV [28] or organ transplant recipients further contributes to resistance [29]. The increasing number of immunocompromised individuals has led to a rise in NAC infections, which are less responsive to fluconazole. VVC can present in acute or chronic forms, significantly affecting women’s quality of life by causing persistent discomfort, stress, and disruption to daily activities and sexual health. The primary reason for focusing on *C. tropicalis* and *C. krusei* in addition to *C. albicans* is their notable resistance to common antifungal treatments, particularly azoles. This resistance can result in treatment failures and recurrent infections. Although *C. krusei* is less prevalent, it poses a significant risk to patients with hematological malignancies, bloodstream infections, and those undergoing bone marrow transplants. Its inherent resistance to fluconazole makes *C. krusei* particularly challenging to treat [30].

Currently, there are around 80 commercially available peptides, with an estimated 500 in clinical development [6,7] and 400–600 in preclinical studies against *Candida* and other pathogens. These peptides are advanced to clinical trials with the following characteristics: superior or equivalent efficacy to existing drugs, favourable tolerability, pharmacodynamics, and pharmacokinetics with minimized side effects. All those characteristics were imparted to natural AMPs by engineering their amino acid sequences, and approximately 90% of the stringent requirements were met. In a clinical setting, 20% of the globally available AMPs have entered clinical trials against candidiasis [31]. CGA-N46, Novexatin, HLR1r, Omiganan, PAC-113, PL-18, and CZEN-002 are a few that are in clinical trial phases for anti-*Candida* drugs. Other than clinical trials, gomesin, gramicidin D and S, bacitracin, polymyxin B, colistin, heliomycin derivative ETD151, lycosin, and daptomycin have shown in vitro activity against fluconazole-resistant [7,31]. *Candida* spp. Nisin is used for food storage purposes [6].

Antimicrobial peptides (AMPs) are short peptides; some of which exhibit helical or beta-sheet structures. These structural properties impart specific characteristics such as the ability to penetrate cells [14] and kill pathogens by forming aggregates [22,32,33,34,35,36,37,38]. The effectiveness of AMPs is influenced by factors such as net charge, hydrophobicity, and amphipathicity, with the amino acid composition being a critical determinant. Additionally, the helical structures of AMPs play a vital role in penetrating the fungal membrane, as emphasized by Sonesson et al. (2007) [39]. *Candida* spp. rely on their cell walls, comprising components such as β-glucan, chitin, and phosphomannoproteins, for protection against environmental stress and antifungal drugs. These components have a negative charge, which makes them potential binding sites for positively charged AMPs. This interaction has been demonstrated in studies like that of Harris, M. et al. (2009) [40], where glycosylation knockouts of *C. albicans* resulted in a reduced negative charge and lower susceptibility to AMPs. It was observed that the binding of certain AMPs to the cell wall is crucial for their antifungal activity and here the charge and hydrophobicity are key factors in their function. Strategic design, guided by sequence databases, can be employed to engineer AMPs with enhanced specificity against microbes and reduced hemolytic activity. These tailored AMPs may be more appropriate for clinical applications. Within this realm, two variants of epinecidin-1, featuring lysine substitutions, have been developed, demonstrating increased lytic activity against *Candida* spp.

Epinecidin-1 inhibited the growth of *C. albicans* and *C. krusei* at 128 μg mL^−1^, and *C. tropicalis* at a minimum inhibitory concentration (MIC) of 256 μg mL^−1^. The histidine-to-lysine substituted variant, variant-1, inhibited all three *Candida* spp. at 64 μg mL^−1^, demonstrating a two-fold increase in activity against *C. albicans* and *C. krusei*, and a four-fold increase for *C. tropicalis*. The improved efficacy may be attributed to an increased helical propensity and a higher charge relative to epinecidin-1. These findings align with prior research on modified peptides, such as C3a peptides [35], the N-terminal domain of bovine lactoferrin [12], uperin 3.6 peptide [41], protamine [42] synthetic helical peptides [22], and KABT-AMP [5], which underscore similar structural features enhancing antimicrobial activity.

The variant-2 generated by the replacement of weak hydrophobic alanine at the thirteenth position of variant-1 with charged amino acid lysine inhibited the growth of *C. albicans* and *C. tropicalis* at 32 μg mL^−1^ and *C. krusei* at 64 μg mL^−1^. For *C. albicans*, there was a four-fold enhanced activity compared to epinecidin-1 and a two-fold enhanced activity compared to variant-1. For *C. tropicalis* there was an eight-fold enhanced activity compared to epinecidin-1 and a two-fold enhanced activity compared to variant-1. For *C. krusei*, there were two-fold enhancements compared to epinecidin-1 and similar for variant-1. The culminated enhanced activity of variant-2 would be from a large patch of positive charge formed by local electrostatic interactions [5,14] that mediate strong binding to cell membranes required for anti-*Candida* activity. 

The majority of the pathogenic *Candida* spp. are protected by the formation of biofilms. During biofilm formation, an extracellular matrix is formed, encasing the microbial cell that prevents the antimicrobial substances from reaching the cells [43,44,45]. Biofilms contribute significantly to microbe’s resistance against antifungal agents; disrupting them could enhance treatment effectiveness [7,23,31,42,43]. Cells associated with biofilm formation require a 1000-fold greater concentration of fluconazole and amphotericin B to inhibit *Candida* spp. biofilm than the planktonically grown strains [44,45]. This feature plays a major role in the persistent re-occurrence of infections and antibiotic resistance. Strikingly, for the in vitro peptide-treated *Candida* spp. cells, the numbers are reduced compared to the control. For the controls a large cluster of *Candida* spp. cells, sticking to the microscopic coverglass inserts are seen as shown in Figure 2. For the peptide-treated *Candida* cells, the numbers were reduced and the cells did not adhere to the surface. These results show that the peptides interfere in biofilm formation. Observing that the AMPs have lytic activity and did not permit the *Candida* spp. cells to adhere on the surface, we postulated that the AMPs may bind to the cell surface glyco-proteins responsible for adhering to the host cells to form biofilm and inhibit the yeast to hyphal transition which is involved in the invasion of host mucosal epithelial cell and tissue damage. Hence, we computationally predicted the binding sites of the peptides to Exo-β-(1,3)-Glucanase, Secreted aspartic proteinase (Sap) 1, and N-terminal domain of *C. albicans* Als9-2 which are involved in the stages of biofilm formation (adherence, growth and proliferation, differentiation and matrix formation and dispersion) based on previous review reports [45,46]

Exo-β-(1,3)-glucanase enzyme is a virulence factor that degrades the *Candida* β-1,3-glucans in the cell wall which helps to invade host tissues and evade the immune system. It also reshapes the *Candida* cell wall for infecting the host cells. Secreted aspartic proteinases (SAPs) are a group of enzymes that play a critical role in the virulence and pathogenicity of *Candida* spp. They are responsible for nutrient acquisition and tissue penetration. Agglutinin-like Sequence 9 (ALS 9) is a group of *Candida* adhesin superfamily that play an important role in adhesion, biofilm formation, and immune evasion leading to virulence of host cells [46].

Computational prediction shows that peptides have a strong affinity to *Candida* spp. cell wall proteins which may in turn block the pathogen from binding to the host cells which was shown in vitro experiments for LL-37, hBD-3 [47], NaD1 [48] and psoriasin [43]. The studied epi-1 and its variant peptides have unique binding sites that interact with specific amino acids to disrupt the biofilm formation at varying degrees.

After determining that the AMPs inhibit the growth of *Candida* spp. cells, the mechanism of disruption was analyzed by scanning electron microscopy (SEM). It shows that the peptide-treated *Candida* spp. cells have pores on the membrane. This may lead to the loss of fungal cytoplasmic contents including metabolite and ions by forming deep pits. Further, the loss of turgor and transmembrane potential were the likely causes of cell shrinkage [49,50]. After confirming that the peptides penetrated through the membrane, their ability to induce Reactive Oxygen Species (ROS) was tested against *C. albicans.* The *C. albicans* was incubated with peptides for 24 h. At this point, the peptides killed the *Candida* spp., and they did not stick to the plates because their adherence potential was disrupted. However, for the leftover adherent *Candida* spp. cells, there was more ROS generation as seen by the increased green fluorescence of DCHFDA which was not seen in the control. The epinecidin-1 and its variants induce ROS generation like other AMPs previously reported from plant defensin [51]. Since the peptides form pores, this will lead to exposure to the oxygenic environment, which increases the ROS in the damaged membrane.

The present study demonstrates the efficacy of the antimicrobial peptide epinecidin-1 and its variants against *Candida* spp. pathogens bioactivity and biofilm formation. Further characterization is required to find the mode of action at a molecular level with and without antifungal agents, which are under pipeline in our laboratory.

## 5. Conclusions

This is the first report on lysine-substituted variants of epinecidin-1 demonstrating anti-*Candida* and antibiofilm effects on multi-drug-resistant clinical isolates. The variants showed two-to eight-fold enhanced activity compared to the wild-type epinecidin-1 and demonstrated potency in destroying membrane integrity and preventing biofilm formation. These quantitative attributes prove the potential application of peptides as anti-biofilm agents on medical implants.

## Figures and Tables

**Table 1:** Sequence of epinecidin-1 and its variants. Lysine-replaced residues are highlighted, bold, italicized, and underlined.

Peptide Name	Sequence
Epinecidin-1	GFIFHIIKGLFHAGKMIHGLV
Variant 1- Replacement of H with K	GFIF***K***IIKGLF***K***AGKMI***K***GLV
Variant 2- Replacement of A with K	GFIFKIIKGLFK***K***GKMIKGLV

**Table 2:** Interface area score of the peptides binding to selected *Candida* membrane proteins (a) Exo-B-(1,3)-Glucanase, (b) Secreted aspartic proteinase (Sap) 1 and (c) N-terminal domain of Als 9-2 responsible for biofilm formation.

	*Candida* Protein Name	PDB ID	Epinecidin-1	Variant-1	Variant-2
a	Exo-B-(1,3)-Glucanase	1CZ1	1065	1338 ↑↑	1298 ↑
b	Secreted aspartic proteinase	2QZW	1370	1286 ↓	1470 ↑
c	N-terminal domain of Als 9-2	2Y7L	1114	1207 ↑	1100 ↓

The up arrow indicates higher interaction scores, while the down arrow indicates lower interaction scores compared to epi-1.

## Data Availability

All data are mentioned in the document and Supplementary Materials.

## Supplementary Materials

The following supporting information can be downloaded at: https://www.mdpi.com/article/10.3390/idr16060096/s1, Table S1: Amino acids of the *Candida* surface proteins that interacts with the peptides. The amino acids of the *Candida* membrane proteins that exhibit shared interactions with the peptides are indicated in bold. As the variants have small MIC values, their interaction with the membrane proteins are strong. Figure S1: Output of imageJ quantitated for number of cells in the fields. Figure S2: Output of imageJ quantitated for number of cells in the fields using binary and threshold settings. The output of results obtained for blue and green channel which were used to calculate the percentage of ROS are shown.

## Abbreviations

ABC	ATP Binding Cassette
AMP	Anti microbial Peptide
CDC	Centre for Disease Control
CFU	Colony Forming Units
DCFHDA	2′,7′-dichlorofluorescein diacetate
MIC	Minimum Inhibitory Concentration
PBS	Phosphate-Buffered Saline
ROS	Reactive Oxygen Species
SSA	Stress-Seventy subfamily A
