# Supplementary material for: Antibiofilm Activity of Epinecidin-1 and Its Variants Against Drug-Resistant Candida krusei and Candida tropicalis Isolates from Vaginal Candidiasis Patients
Source: Infect Dis Rep. 2024 Dec 12;16(6):1214–29. doi: 10.3390/idr16060096 (PMC11675185; doi:10.3390/idr16060096)

Supplementary Table 1. Amino acids of the *Candida* surface proteins that interacts with the peptides. The amino acids of the *Candida* membrane proteins that exhibit shared interactions with the peptides are indicated in bold. As the variants have small MIC values, their interaction with the membrane proteins are stronger.

	***Candida* protein Name**	**PDB ID**	**Epi**	**Var-1**	**Var-2**
a	Exo-B-(1,3)-Glucanase	1CZ1	VAL 4<br>TRP 8<br>ARG 16<br>ASN 126<br>ASN 127<br>THR 357	ASP 151<br>TYR 153<br>VAL 197<br>PHE 229<br>**GLN 230**<br>**PHE 232**<br>GLU 262<br>ASP 280<br>ASN 305<br>ARG 309	PRO 196<br>**GLN 230**<br>VAL 231<br>**PHE 232**<br>ARG 309<br>TYR 317<br>ASP 318
b	Secreted aspartic proteinase	2QZW	ARG 54<br>GLY 220<br>**THR 221**<br>THR 222<br>**TYR 225**<br>GLN 228<br>**HIS 249**<br>TYR 252<br>SER 300<br>ILE 301	LYS 49<br>GLY 85<br>ASP 86<br>GLY 87	HIS 11<br>VAL 12<br>**THR 221**<br>**TYR 225**<br>GLY 248<br>**HIS 249**<br>GLU 278<br>ALA 282<br>GLN 295<br>LEU 298<br>ARG 312
c	N-terminal domain of Als 9-2	2Y7L	TYR 21<br>VAL 22<br>GLY 165<br>TYR 166<br>CYS 188<br>GLU 189<br>CYS 280<br>GLY 283<br>ASN 284<br>ASP 288<br>THR 291	**THR 63**<br>**THR 65**<br>ASN 82<br>PHE 87<br>PRO 174<br>**SER 175**<br>ASN 177<br>**ILE 267**<br>ASN 299	**THR 63**<br>**THR 65**<br>ASP 80<br>SER 92<br>**SER 175**<br>GLU 265<br>**ILE 267**<br>ASP 268

Supplementary figure.1. Output of imageJ quantitated for number of cells in the fields.

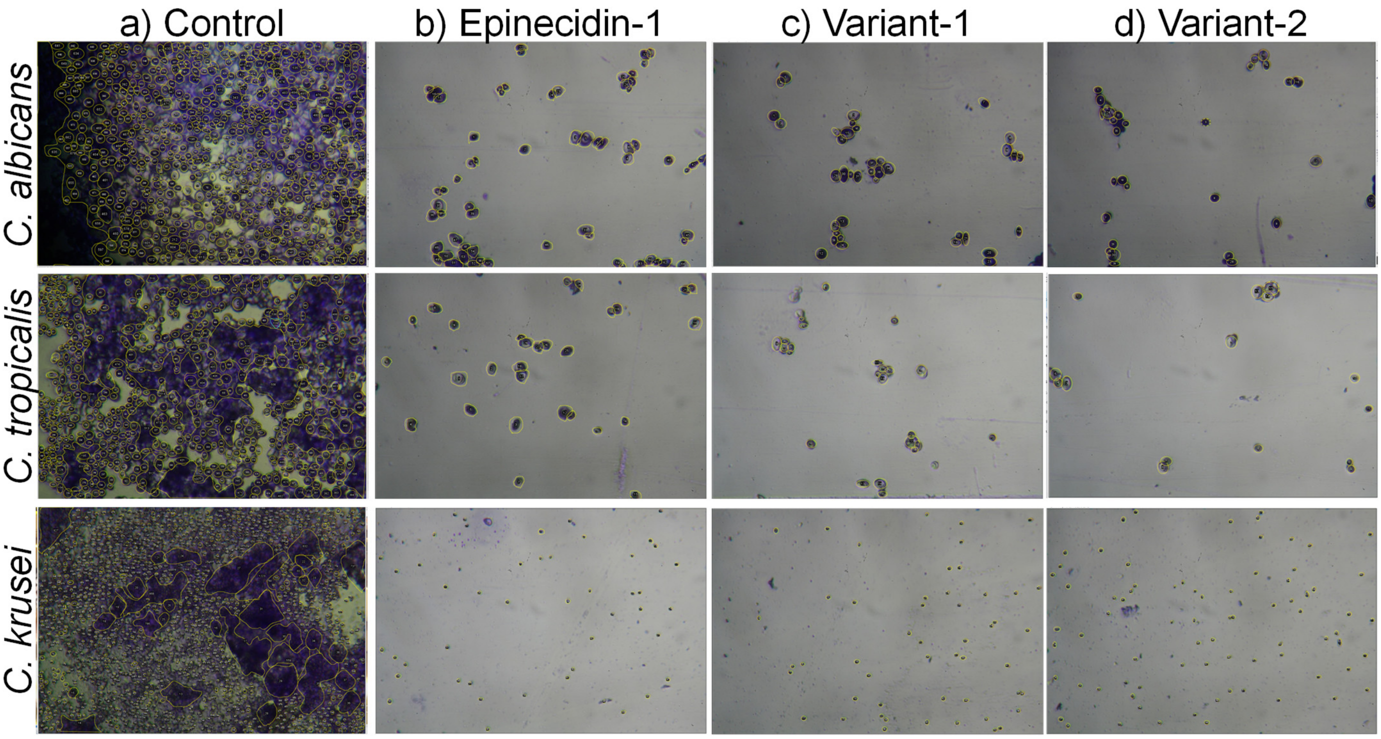

Supplementary figure.2. Output of imageJ quantitated for number of cells in the fields using binary and threshold settings. The output of results obtained for blue and green channel which were used to calculate the percentage of ROS are shown.

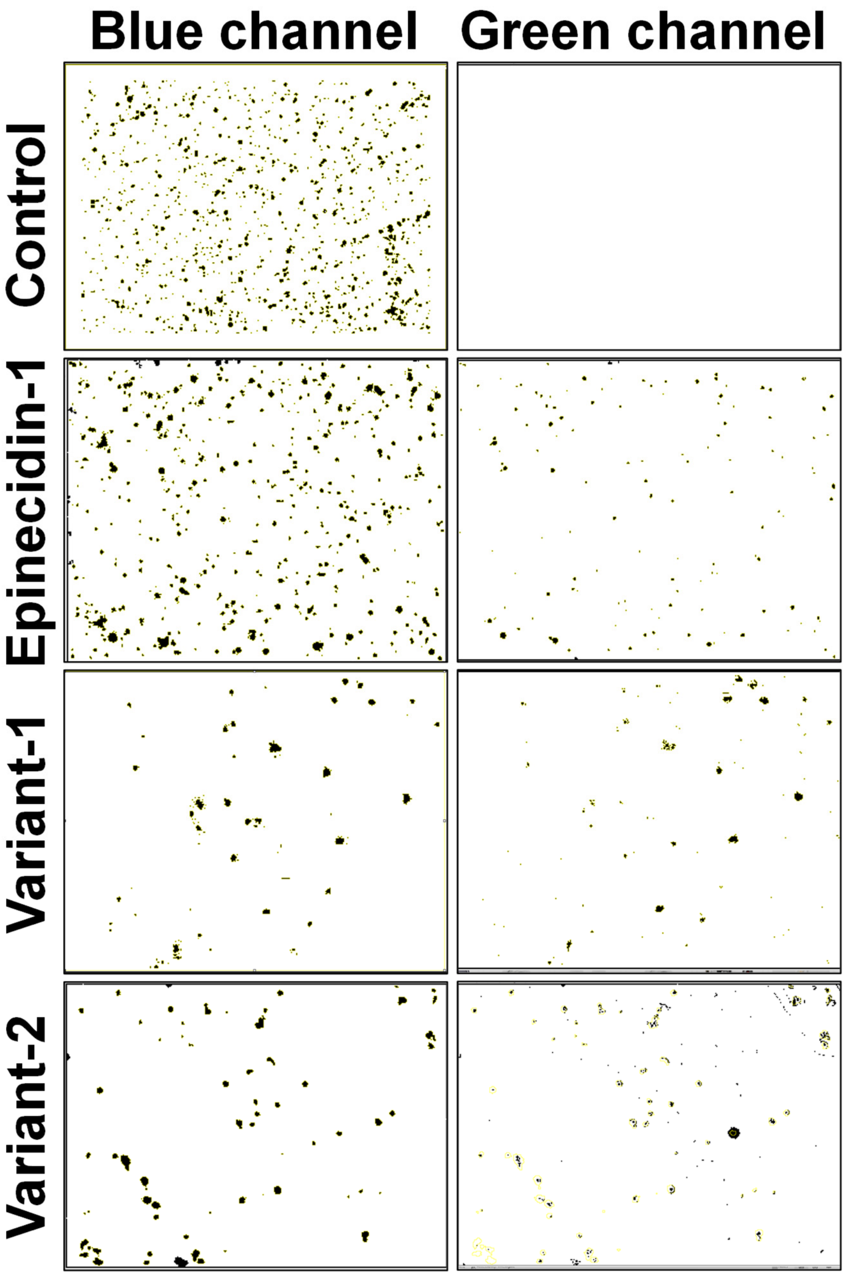

Supplement: Supplementary file 1 [file idr-16-00096-s001.zip › idr-3236227-supplementary.pdf]
